# The coupling relationship and driving mechanism between urbanization and ecosystem services in the Yellow River Basin from a multi-spatial scale perspective

**DOI:** 10.1371/journal.pone.0293319

**Published:** 2023-12-07

**Authors:** Jinyuan Zhang, Zhongwu Zhang, Liping Liu, Xue Bai, Shiyu Wang, Lei Kang, Xingran Cai

**Affiliations:** 1 College of Geographic Science, Shanxi Normal University, Taiyuan, China; 2 Institute of Human Geography, Institute of Ecology and Environment of Yellow River Basin, Taiyuan, China; Sichuan University, CHINA

## Abstract

Rapid urbanization has led to ecological destruction and associated issues. Macro policies wield substantial influence over urbanization and its relationship with the environment. Without considering the differences in scale, macro policies may be ineffective at addressing urbanization’s adverse impacts on the environment, and even worsen this relationship. We used data on 622 counties, 76 prefectures, and 7 urban agglomerations in the Yellow River Basin to examine the development level, coupling coordination degree, and spatial patterns of urbanization and ecosystem services at three scales during 2000–2020. Further, we explored the driving mechanisms in the relationship between urbanization and ecosystem services. We found that: First, the coupling coordination was relatively low but showed an upward trend. A sizeable spatiotemporal difference existed, with higher (lower) coordination in the east (west). Second, the coupling coordination of each scale exhibited significant spatial positive correlations. The low-value heterogeneous region was embedded around the agglomeration region, and polarization was significant. The larger the scale, the stronger the agglomeration effect. Further, the coupling coordination spatial agglomeration effect of each scale gradually weakened over time. Third, the spatial and temporal distributions of coupling coordination and its agglomeration characteristics at different scales differed. The urban agglomeration scale showed significant overall coordination or agglomeration characteristics, and prefecture and county regions showed local and unique characteristics within urban agglomerations. Fourth, the dominant factors influencing the spatial patterns of the coupling coordination at the county, prefecture, and urban agglomeration scales differed. The interaction and factor detection showed linear and double-factor enhancements. We find that economic development, government policies, environmental protection, and natural factors are the combined effects of urbanization and ecosystem services. Our research method can provide a reference for other river basins, and the results can help governments in formulating policies for sustainable development at different spatial scales.

## 1 Introduction

Urban expansion and population agglomeration caused by rapid urbanization cause significant changes in the structure and function of ecosystems [1]. Urbanization can bring cities closer, eventually giving rise to urban agglomerations. As the optimal form of spatial organization for urban development, urban agglomerations are a collection of economic agglomerations characterized by rapid industrialization [2]. However, the resulting intensification of human activities brought about by the concentration of urban agglomerations has placed tremendous pressures on the ecological environments in urban agglomerations, and those in surrounding counties and cities. These pressures can lead to the loss of arable land [3], reduced carbon stocks [4], and climate change [5], further increasing the risk of ecosystem stress and environmental degradation, and threatening regional sustainable development [6]. Essentially, urbanization and ecosystem services exhibit a complex coupling relationship. As such, a key research issue is how should we coordinate this relationship.

In highly urbanized areas, human activities are the dominant factor affecting the functioning of urban ecosystems. Scholars have discussed the relationship between the two at the watershed, province, prefecture, and county scales [7, 8], and studied ecosystem services [9], land economic density [10], and human well-being [11] in a single spatial environment. Single-scale research must compare multi-system relationships from multiple spatial perspectives of the same element.

Among studies quantifying the relationship between urbanization and ecosystem services, Fang et al. [12] theorized the interactive coupling effect between urbanization and the ecosystem. Liu et al. [13] proposed the concept of "coupling Rubik’s cube" to explain the coupling mechanism between urbanization and ecological environment from the four dimensions of space, time, appearance, and organization. Many studies have quantified the relationship between urbanization and ecosystem services using multi-source data [14], such as yearbook, night light, and ecological index remote sensing data [15]. However, there are few regional parameters which quantitatively measure ecosystem service value at the watershed level [16]. Here, we use the InVEST model to quantitatively measure ecosystem service capacity. After formulating model coefficients according to regional characteristics, we fit the real data for simulation and use the coupling coordination model to quantitatively evaluate the relationship between the two system elements.

Research on the driving mechanisms of coupling and coordination relationships has mainly adopted methods such as data spatial superposition [17], the STIRPAT model [18], the system dynamics (SD) model [19], the interactive stress model [20], geographical detector [21], and the geographically weighted regression series model [22], among others. These studies show that urbanization and ecological services can interact during its rapid development stage, urbanization significantly impacts the ecological environment. Macro policies to regulate the city can effectively reduce ecological pressures. However, extant research only identifies single factors. Moreover, macro policies do not consider the differences in scale, and consequently, may be ineffective or even worsen the relationship between urbanization and the ecological environment.

In China, the Yellow River Basin (YRB) is an essential experimental area for developing an ecological environment, with ecological protection regarded as an important project. However, balancing urbanization and ecosystem services require better natural conditions and more substantial carrying capacity [23]. In 2022, the urbanization rate in the YRB was 61.30%, which is approximately 3.9 percentage points lower than the national average. The proportion of built-up area of core cities in each province is increasing, while the areas of forests and water bodies, and natural ecosystem are gradually shrinking. In particular, in the middle and lower reaches of the YRB, soil erosion and ecological damage are severe, yet urban development continues. As such, the imbalance between urban and ecological development in all provinces and regions along the YRB needs to be solved urgently [24, 25]. Studies have theoretically discussed the scenario and intensity of the interaction between urbanization and ecosystem services in the YRB to guide regional urbanization and sustainable development of the ecological environment [26]. In addition, different urban development policies at different scales have led to deterioration of the ecological environment. Therefore, we need a better explanation of the coupling relationship and change rules between various factors of urbanization and ecosystem services in the YRB from multiple spatial scales.

This study quantifies urbanization and ecosystem services from a multi-spatial scale perspective using data on 622 counties, 76 prefectures, and 7 urban agglomerations in the YRB, and analyzes the coupling coordination degree (CCD) between urbanization and ecosystem services. Further, we study the dominant factors affecting CCD at different spatial scales. Our specific objectives are as follows: (1) To quantify and map the urbanization and ecosystem service levels at the county, prefecture, and urban agglomeration scales in 2000, 2005, 2010, 2015, and 2020 using the coefficient of variation method; (2) Use the coupling coordination model to detect the CCD between urbanization and ecosystem services at the three scales, and use the spatial autocorrelation method explore the spatial agglomeration degree; (3) Identify the dominant factors affecting the CCD at the three scales using factor and interactive detections through the geographic detector model.

This study analyzed and explored the development laws of coupling coordination, explored the influencing factors of coupling coordination from multiple spatial perspectives, and provided decision-making basis for the coordinated development of the two systems. The research helps to provide reference for other watersheds, and the research results also provide a theoretical basis for relevant governments in the Yellow River Basin to formulate urban development and environmental protection policies at different spatial scales.

## 2 Overview of the study area

**Fig 1:**
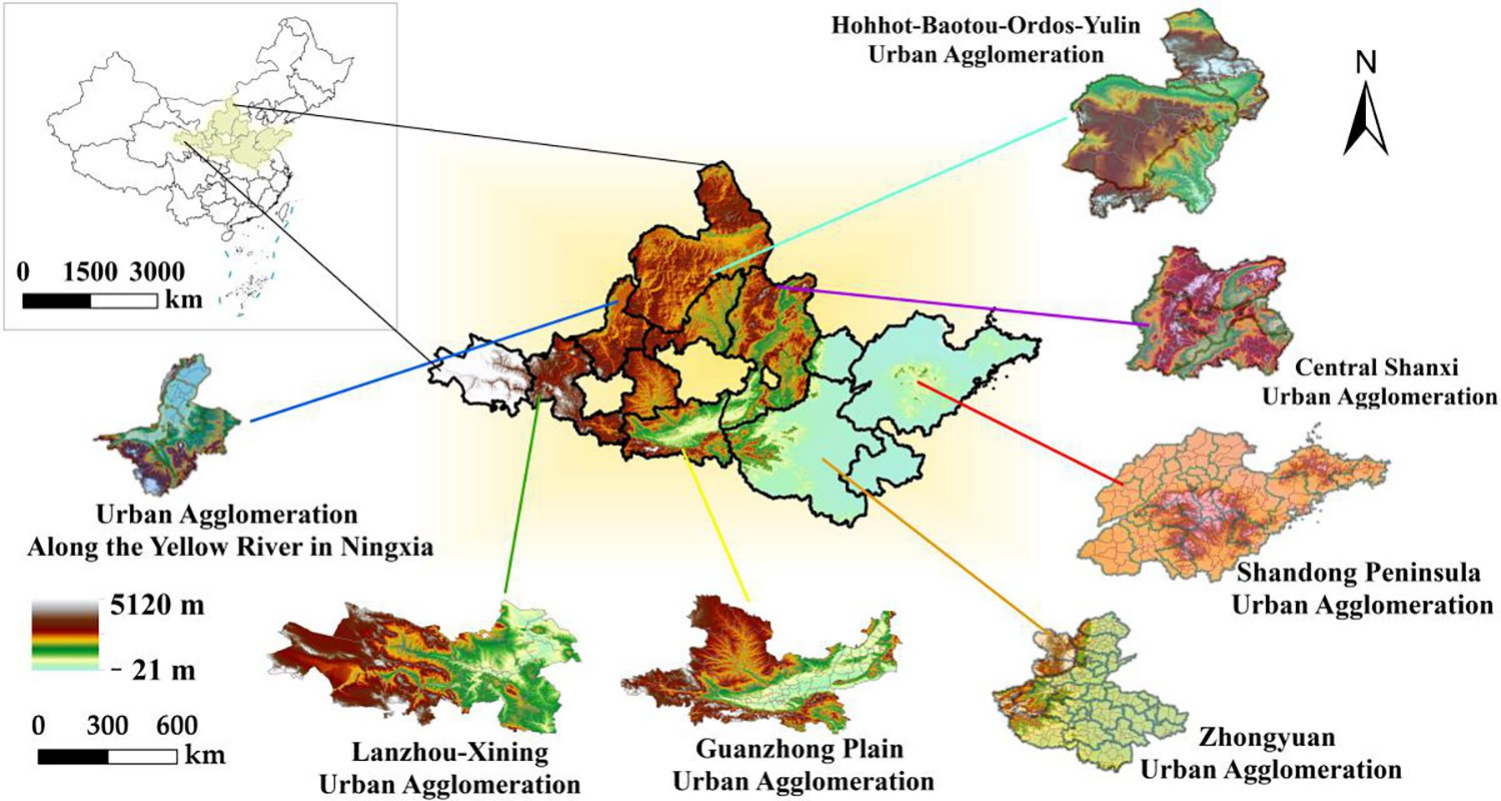
Seven urban agglomerations in the YRB.

The YRB is an essential ecological reserve and economic zone in China, involving nine provinces (including autonomous regions), including Qinghai, Gansu, Sichuan, Ningxia, Inner Mongolia, Shaanxi, Shanxi, Henan, and Shandong, and seven urban agglomerations, including the Shandong Peninsula, Central Plain, Central Shanxi, Huhhot-Baotou-Ordos-Yulin, Guanzhong Plain, Ningxia, and Lanzhou-Xining urban agglomerations. It includes 76 prefectures and 622 counties (Fig 1) and is located at 32°6′53″N~41°48′18″N, 95°50′29″E~119°06′53″E, with a total length of 5464 km and a basin area of 3.6×10^6^ km^2^. The average annual precipitation in the whole basin is 466 mm. The area of soil and water loss in the Loess Plateau is 43.4×10^4^ km^2^, of which the area with an annual average erosion modulus of more than 5000t/km^2^ is approximately 1.56×10^4^ km^2^. The average annual natural runoff of the entire river is 58 billion m^3^, accounting for only 2% of the total river runoff in China [23, 25].

Significant economic differences exist in the YRB. In 2021, the GDP of Shandong province exceeded 8 trillion yuan, ranking third in China, while the GDP of Ningxia and Qinghai were only 452.23 and 334.66 billion yuan, respectively [23]. The urbanization rate in the YRB (61.30%) is approximately 3.9 percentage points lower than the national average (65.20%), indicating significant regional differences in urbanization. The total land use area of the YRB is 2.03×10^6^ km^2^, with grasslands accounting for the most at 41.47% with an area of 8.42×10^5^ km^2^. The second common land use is unused land, accounting for 25.86% and an area of 5.25×10^5^ km^2^. The YRB comprises complex and diverse ecosystem types, fragile ecological environments, and complex landforms [7]. As such, protecting the ecological environment together with rapid urbanization has become essential for high-quality development balanced by ecological protection in the YRB [27].

## 3 Methodology

### 3.1 Data sources

The urbanization rate data of 622 counties, 76 prefectures, and 7 urban agglomerations in the YRB in 2000, 2005, 2010, 2015, and 2020 are extracted from the "Statistical Yearbook," "City Yearbook," and statistical bulletins of provinces and cities in the corresponding years.

Data such as the digital elevation model (resolution of 90 m), land use data (resolution of 30 m), 1 km population density, and GDP density data are from the Resource and Environmental Science Data Center of the Chinese Academy of Sciences. PM2.5 data are collected from the Atmospheric Meteorological Analysis Group of Dalhousie University (http://fizz.phys.dal.ca/). CO_2_ data are from the National Geophysical Data Center (http://www.ngdc.noaa.gov/). The administrative boundary map is extracted from the standard map service website of the Ministry of Natural Resources (http://bzdt.ch.mnr.gov.cn/). Road network traffic data are from the public street map platform (http://www.openstreetmap.org/).

Meteorological data, such as precipitation and evapotranspiration, are from the National Meteorological Science Data Center (http://data.cma.cn/). Data on soil type, soil organic matter content, and root depth are from the National Scientific Data Research Center for the Qinghai-Tibet Plateau (http://data.tpdc.ac.cn/). The unified projection coordinates of all raster data are WGS_1984_World_Mercator. According to the InVEST model manual, we export the unified raster data resolution of 300 m to TIF format data and the InVEST model is added to estimate the water production service. This yields the new raster data. Then, the unified zoning statistics are performed to obtain the raster quantification in each county, prefecture, and urban agglomeration unit.

### 3.2 Comprehensive urbanization rate

Following previous studies [11, 14], we construct the comprehensive urbanization rate of the YRB from the dimensions of population, economy, land, and society (**Table 1**). We divide the development level of urbanization into four stages: germination [0, 0.10) initial [0.10, 0.25), rapid development [0.25, 0.50), and highly developed stages [0.50, 1.00].

**Table 1:** Comprehensive urbanization weight of the YRB.

Indicators	Secondary Indicators	County	Prefecture	Urban Agglomerations
Population urbanization	Urbanization rate of resident population (%) X_1_	0.055	0.041	0.016
	Population density (person/km^2^) X_2_	0.085	0.116	0.091
Economic urbanization	GDP Growth Rate (%) X_3_	0.181	0.096	0.067
	Added value of the tertiary industry (CNY) X_4_	0.021	0.017	0.014
	GDP density (10^4^CNY/km^2^) X_5_	0.148	0.162	0.126
Land-use urbanization	Proportion of urban built-up area in the total area of a city (%) X_6_	0.151	0.157	0.189
	Urban spatial expansion intensity (%) X_7_	0.146	0.147	0.147
	Urban impervious surface area (km^2^) X_8_	0.117	0.147	0.209
Social urbanization	Number of hospital beds (beds) X_9_	0.096	0.117	0.141

We use the coefficient of variation method to calculate the index weight [28]:

$$W_{i}=V_{i}/\sum_{i=1}^{n}V_{i}\;V_{i}=\frac{S_{i}}{\bar{A_{i}}}(i=1,2,\ldots ,n)$$ (1)

*W*_*i*_ is the weight of the *i* th index, and $V_{i}、S_{i}、\bar{A_{i}}、W_{i}$ are the coefficient of variation, standard deviation, and average of the *i* th index, respectively.

### 3.3 Quantifying ecosystem services

We use the InVEST model to assess ecosystem services in the YRB considering region-specific parameters of the actual ecosystem. Following relevant studies on ecosystem services [29, 30], we use five modules to evaluate the ecosystem service capacity of the YRB: carbon sequestration service (Y_1_), habitat quality (Y_2_), soil conservation (Y_3_), water conservation (Y_4_), and food supply (Y_5_) (**Table 2**). Then, following existing studies [31], we use SPSS 27 and the K-means clustering method to classify the ecosystem service capability of the YRB into four categories: primary (0.00, 0.15), intermediate [0.15, 0.39), intermediate, advanced [0.39, 0.53) and advanced [0.53, 1.00).

**Table 2:** Evaluation system of ecosystem services in the YRB.

Ecosystem services	County	Prefecture	Urban Agglomeration
Carbon sequestration Y_1_	0.137	0.145	0.129
Habitat quality Y_2_	0.247	0.258	0.137
Soil conservation Y_3_	0.252	0.235	0.242
Water conservation Y_4_	0.142	0.195	0.326
Grain supply Y_5_	0.222	0.167	0.166

#### 3.3.1 Carbon sequestration

The carbon sequestration parameters of different land use types are quantified using the InVEST model carbon module [32]:

$$C_{x}=C_{above}+C_{below}+C_{soil}+C_{dead}$$ (2)

where *C*_*x*_ is the carbon sequestration of grid unit *x* (t/hm^2^), *C*_*above*_ is the carbon stored in the aboveground biomass, *C*_*below*_ is the carbon stored in the underground biomass, *C*_*soil*_ is the carbon stored in the soil, and *C*_*dead*_ is the carbon stored in dead organic matter.

#### 3.3.2 Habitat quality

The habitat quality module of the InVEST model is used to quantify the threat and sensitivity characteristics, degree of habitat degradation, and habitat quality under spatial and temporal changes [33].

$$Q_{xj}=H_{j}(1-(\frac{D_{xj}^{2}}{D_{xj}^{2}+k^{2}}))$$ (3)

where *H*_*j*_ is the habitat suitability of land use type *j*, $D_{xj}^{2}$ is the total threat level of grid pixel *x* in land type *j*, and *k* is a saturation constant.

#### 3.3.3 Soil conservation

The soil conservation module of the InVEST model is based on the soil loss equation. It is calculated based on data such as landform, climate, vegetation, and land management status [34] as follows:

RKLS=R×K×LS,RUSLE=R×K×LS×C×P (4)

SC=RKLS−RUSLE (5)

*RKLS* is potential soil erosion, *RULSE* is substantial soil erosion, *SC* is actual soil conservation (*tkm*^*-2*^*a*^*-1*^), R is the rainfall erosivity factor, and *K* is the soil erodibility factor. *LS* is the slope factor, *L* is the length, and *S* is the slope. *C* is the vegetation management factor, and *p* is the factor of soil and water conservation measures.

#### 3.3.4 Water conservation

The water yield module of InVEST model quantifies the water yield. According to *Budyko* theory, the ratio between the actual evaporation and precipitation is related to potential evapotranspiration [35]. The annual water yield of each pixel *WY* (*x*) on the ground is calculated as follows:

$$WY(x)=(1-\frac{AET(x)}{P(x)})\times P(x)$$ (6)

where *AET(x)* is the actual evapotranspiration of *x* pixel and *P(x)* is the annual precipitation on *x* pixel.

#### 3.3.5 Grain supply

Research shows a significant linear relationship between grain production and the normalized vegetation index. We simulate the service space of grain production in the YRB using statistical data of grain production in the YRB and NDVI data [36] as follows:

$$CP_{x}=\frac{NDVI_{x}}{NDVI_{sum}}\times CP_{sum}$$ (7)

where *CP*_*x*_ is the grain yield of the grid unit (*t*), *NDVI*_*x*_ is the NDVI of the grid unit *x*, *NDVI*_*sum*_ is the normalized difference vegetation index of the entire study area, and *CP*_*sum*_ is the total grain yield of the study area.

### 3.4 Coupling coordination degree model

We compute CCD [15, 16] as follows:

$$C=2\sqrt{u_{1}+u_{2}}/\left(u_{1}+u_{2}\right)$$ (8)

$$D={\left(C+T\right)}^{1/2},T=\partial u_{1}+\beta u_{2}$$ (9)

where *u*_*1*_ and *u*_*2*_ are the urbanization rate and ecosystem service level, respectively; *D* is the CCD; and T is the degree of coordination of urbanization and ecosystem services. *α* and *β* are the contribution of urbanization and ecosystem services, respectively; we set *α* = *β* = 0.5. Following existing research [14, 37], we divide the CCD into severe disorder, mild disorder, moderate, coordination, and superior coordination at intervals of 0.4, 0.6, and 0.7 coordination levels.

### 3.5 Bivariate spatial autocorrelation

We used Moran ’s *I* index to measure global spatial autocorrelation [38]:

$$I=\frac{\sum {}_{i=1}^{n}\sum {}_{j\neq 1}^{n}W_{ij}(x_{i}-\bar{x})(x_{j}-\bar{x})}{S^{2}\sum {}_{i=1}^{n}\sum {}_{i=1}^{n}W_{ij}}$$ (10)

where *i* is the global autocorrelation index; *x*_*i*_ and *x*_*j*_ represent the observed values of units *i* and *j*, respectively; *n* is the number of spatial samples; and *W*_*ij*_ is the spatial weight matrix. Moran’s *I* = 0 indicates that the spatial distribution pattern tends to be random. Moran’s *I* > 0 indicates that space is positively correlated. The larger the value, the more substantial the spatial similarity. LISA is used to measure the local spatial autocorrelation and divided into four types: high-high (H-H), high-low (H-L), low-high (L-H), and low-low (L-L).

### 3.6 Geodetector model

Factor and interaction detections in the geographic detector model [36] are used to identify the influencing factors and their interactions as follows:

$$q=1-\frac{1}{n\sigma^{2}}\sum_{h=1}^{L}n_{h}\sigma_{h}^{2},(h=1,2,\ldots ,L),q\in [0,1]$$ (11)

where *q* is the explanatory degree of each driving factor of urbanization; *n*、*σ*^2^ represent the total sample size and variance, respectively; and *n*_*h*_、*σ*_*h*_ represent the sample size and sample variance of the *h-*th, respectively. The closer the value of *q* is to 1, the greater the influence of this factor on multi-scale CCD, and vice versa.

Interaction detection [21] is the explanatory force value obtained by the coupling coordination degree of two systems under multiple spatial perspectives, which is jointly influenced by two different driving factors. The explanatory power of interactive detection is enhanced or weakened, or the two are independent, compared to the explanatory power of factor detection. There are five main categories of interaction factor detection relationships (**Table 3**).

**Table 3:** Types of interactive detection results for geographic detectors.

Theory of Judgment	Interaction
$q(X_{1}\cap X_{2})<\min(q(X_{1}),q(X_{2}))$	Nonlinear weakening
$\min(q(X_{1}),q(X_{2}))<q(X_{1}\cap X_{2})<\max(q(X_{1}),q(X_{2}))$	Single factor nonlinear weakening
$q(X_{1}\cap X_{2})>\max(q(X_{1}),q(X_{2}))$	Two-factor enhancement
$q(X_{1}\cap X_{2})=q(X_{1})+q(X_{2})$	Independence
$q(X_{1}\cap X_{2})>q(X_{1})+q(X_{2})$	Non-linear enhancement

Following relevant scholars [14, 39, 40], this study considered the CCD between multiscale urbanization and ecosystem services in the YRB as the explained variable. To establish the index, we select explanatory variables from six perspectives: economic development, government behavior, social investment, essential public services, environmental protection, and natural conditions (**Table 4**). We then use a geographical detector to detect the factors and interactions of the CCD between the county, prefecture, and urban agglomeration scales in the YRB.

**Table 4:** Index of influencing factors of coupling coordination spatial pattern of multi-scale two systems.

Independent Variable	Influencing Factors	Variable Explanation
Economic Development	Per capita GDP (CNY) Z_1_	The quality of regional economic development
	Proportion of the added value of the secondary and tertiary industries in GDP (%) Z_2_	Proportion of non-agrarian industries in the industrial structure
	Savings balance of urban and rural residents (CNY) Z_3_	Livelihood between rural and urban areas
Government Implementation	Per capita financial expenditure (CNY) Z_4_	Government regulation ability
Social Investment	Social aggregate investment in fixed assets (CNY) Z_5_	Social investment level
Fundamental Public Service	Number of primary and secondary school students (person) Z_6_	Education Services
	Transit network extent (km) Z_7_	Transport accessibility
Environmental Protection	PM2.5 Concentration (ton) Z_8_	Environmental quality
	CO_2_ emissions (ton) Z_9_	Greenhouse gas emissions
Natural Conditions	Relief intensity (m) Z_10_	Natural Conditions
	Average slope Z_11_	

## 4. Research results and analysis

### 4.1 Spatial distribution characteristics of urbanization and ecosystem services in the YRB

**Fig 2:**
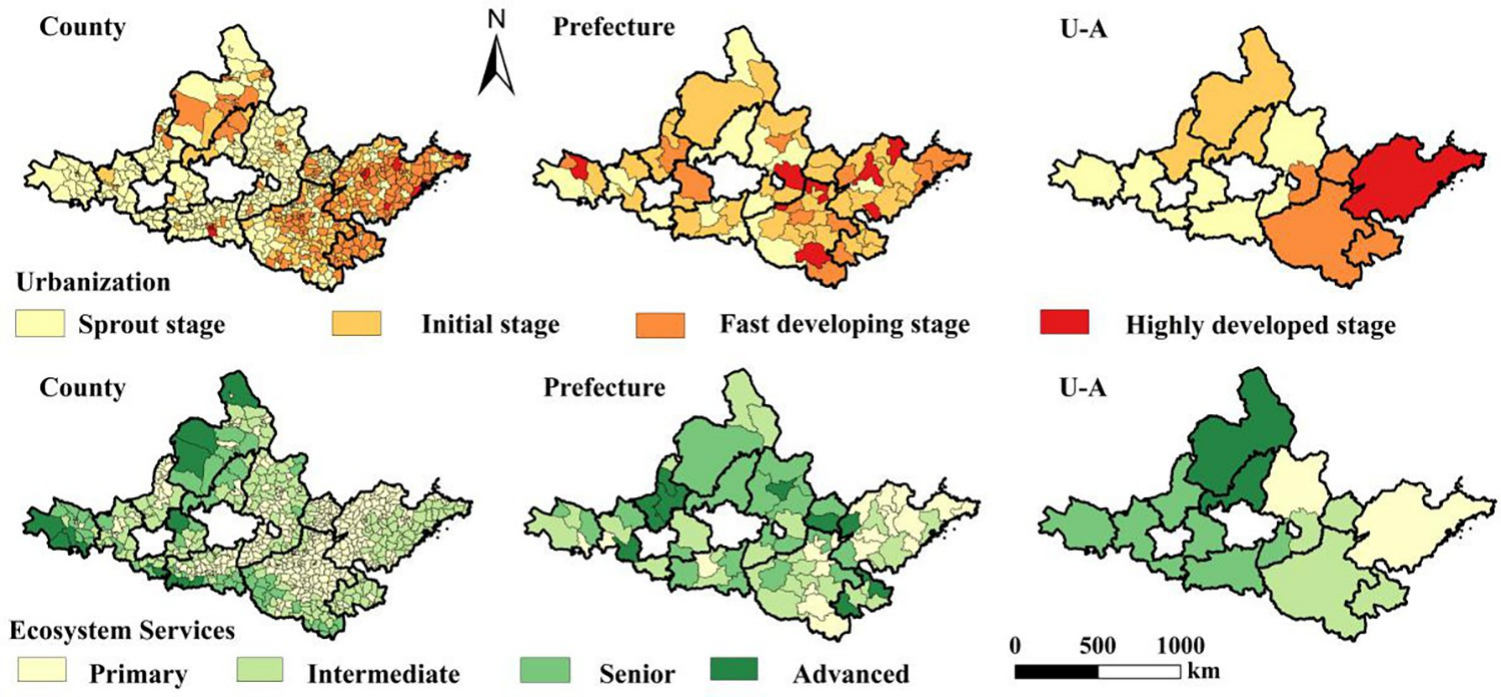
Evaluation of multi-scale urbanization and ecosystem services in the YRB. U-A refers to urban agglomeration.

The urbanization development level in the YRB is relatively low (Fig 2). The number of counties in the rapid and high-speed development stages is small, and they are scattered in the Shandong Peninsula and Central Plains urban agglomerations. Spatially, the urban and urban agglomeration scales are in the budding stage, with a large number of urban agglomerations concentrated in the Lanzhou-Xining, Guanzhong Plain and central Shanxi, Ningxia along the Yellow River, and Huhhot-Baotou-Ordos-Yulin urban agglomerations in the initial stage, and Central Plains and Shandong Peninsula urban agglomerations in the rapid development stage.

The level of ecosystem services is also low. The spatial distribution of ecosystem services is opposite to that of the urbanization development level. The majority of counties are low-level (63.67%). The prefecture scale accounts for 34.21% of the total number of cities, mainly distributed in the Shandong Peninsula and Central Plains urban agglomerations, contrary to the spatial distribution of urbanization levels. This is because the expansion of urban space enhances the intensity of human activities, leading to the destruction of the ecological environment and ecological problems, and reducing the level of ecosystem services.

In the high/low level of overall urbanization and ecosystem service in urban agglomerations, the levels of urbanization and ecosystem services at the prefecture and county scales differ. This indicates that the overall development level should be evaluated from different spatial perspectives. The larger the scale, the more macro the perspective. It does not indicate that the prefecture or county levels are also high CCD at a microscale. This is because the spatial and temporal heterogeneity of the distribution of economics, land, population, and other resources within the prefecture and county, as well as the degree of ecological damage, will lead to changes in the factors used to evaluate urbanization and ecosystem services, thus affecting the overall development level. For example, if the overall urbanization level of the Shandong Peninsula urban agglomeration is relatively high, cities with different levels of urbanization development will exist within it, and the micro-scale in county will once again show different urbanization development levels from the prefecture.

### 4.2 Spatiotemporal pattern of CCD between urbanization and ecosystem services in the YRB

**Fig 3:**
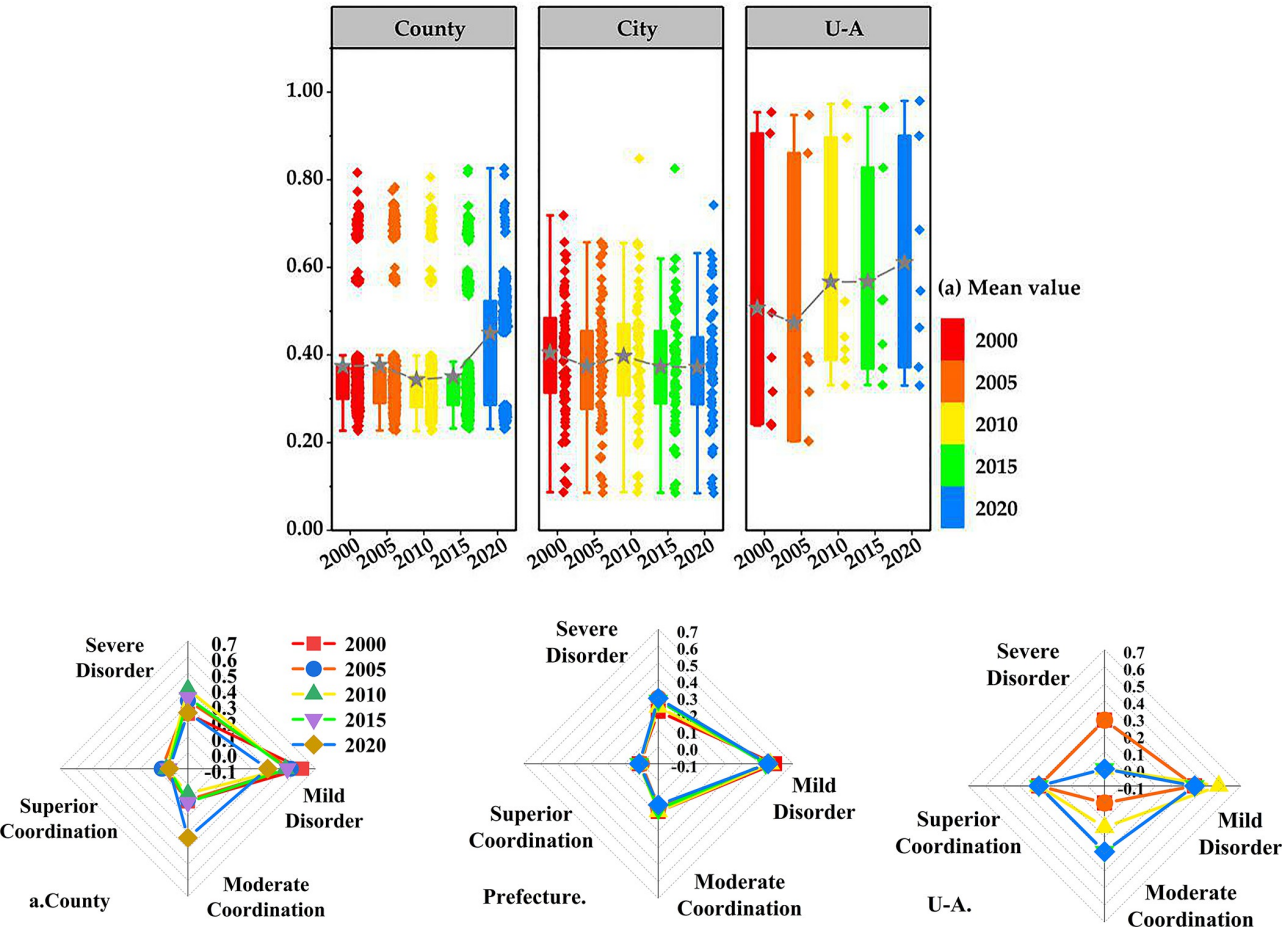
CCD proportion diagram of multi-scale urbanization and ecosystem services in the YRB. U-A refers to urban agglomeration. a) Box plot of the temporal distribution of multi-scale CCD. b) Proportion of the number of samples in each stage of multi-scale CCD.

From the perspective of developmental trends (Fig 3A), the spatiotemporal differences in the CCD at different scales are large and fluctuation characteristics are significant. From 2000 to 2020, the CCD of urbanization and ecosystem services at different scales in the YRB is county (0.37) < prefecture (0.38) < urban agglomeration scales (0.54). The CCD at the county scale increases by 0.07 from 2000 (0.37) to 2020 (0.44) albeit in a U-shaped trend of first decreasing and then increasing. The prefecture scale decreases by 0.03 from 2000 (0.40) to 2020 (0.37). The urban agglomeration scale increases by 0.11 from 2000 (0.50) to 2020 (0.61), showing a "V"-shaped fluctuation. Overall, the CCD between urbanization and ecosystem services at the county and urban agglomeration scales increases in the YRB, peaking in 2020, with an overall stable albeit slight decrease at the prefecture scale. From the location of the numerical concentration of the CCD (box position of box plot), we get: county scale < prefecture scale < urban agglomeration. This indicates that within the same spatial scope, different spatial scales will show different agglomeration characteristics.

From 2000 to 2020, multiple spatial scales were found in the quantity proportion of each coupling coordination stage (Fig 3B). For all the three spatial scales, the number of samples in the moderate coordination stage was the largest, and the quantity proportion showed a trend of decreasing. The number of samples in the moderate coordination stage began to increase in 2010, and the number of samples in the excellent coordination stage accounted for the smallest, which remained basically unchanged. Strong stability. At the county scale, the number of counties in the moderate imbalance stage reached 61.60% (2000), and the number of counties in the moderate imbalance stage decreased to 40.00% in 2020, and the number of counties in the moderate coordination stage increased by 22.72 percentage points from 2000 (10.61%) to 2020 (33.33%). The number of cities in each coupling coordination stage is relatively stable, and the ratio changes little. Due to the relatively large scale and small quantity of urban agglomerations, the number of seriously dysfunctional urban agglomerations has decreased to 0 since 2010, and the proportion of moderately coordinated urban agglomerations has increased by 14.34 percentage points from 2010 (14.33%) to 2020 (28.67%). This indicates that the coupling coordination of urbanization and ecosystem services at different spatial scales in the Yellow River Basin is on the rise from 2000 to 2020, and the smaller the spatial scale, the smaller the mean coupling coordination degree.

**Fig 4:**
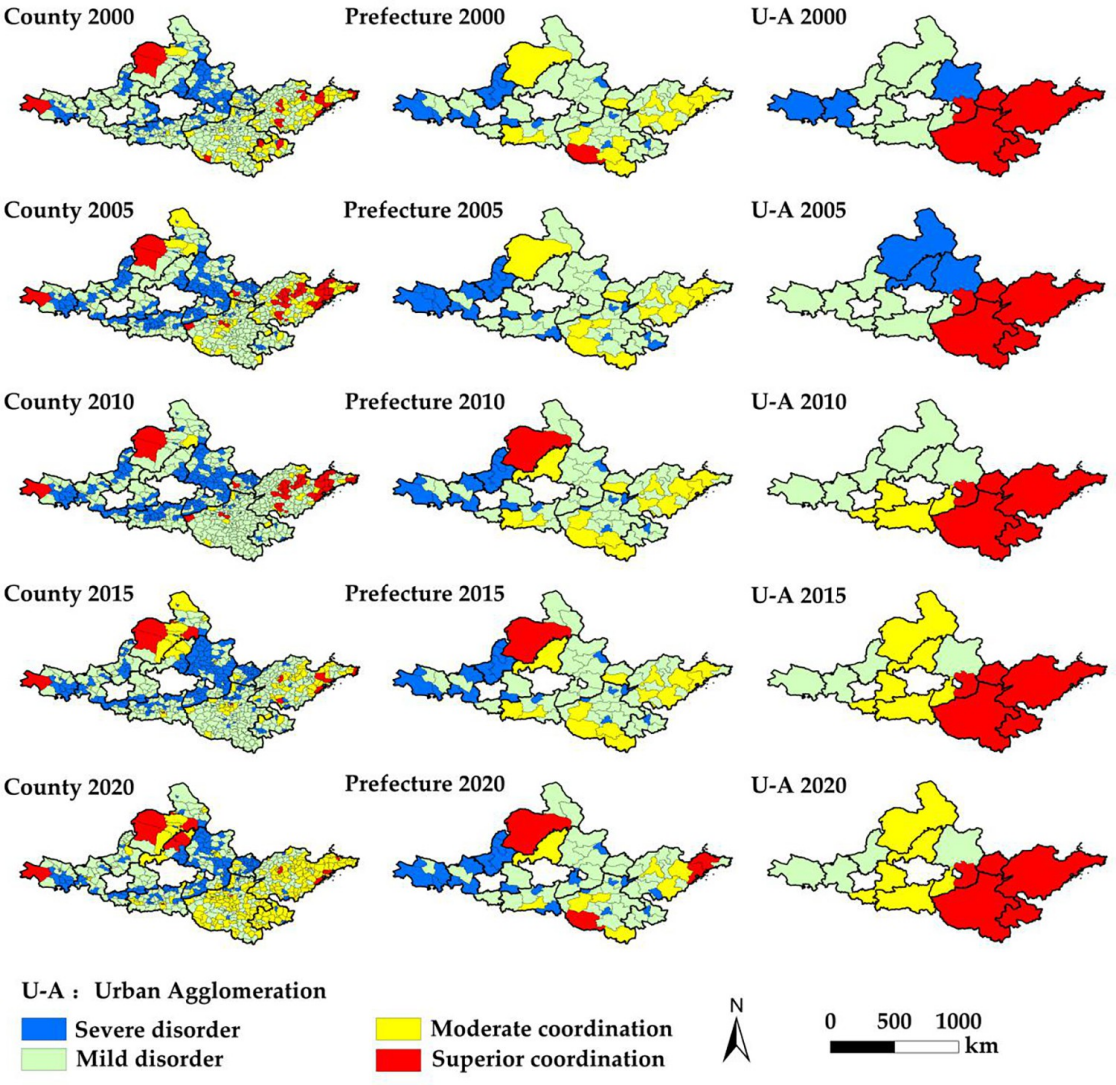
Spatial distribution characteristics of CCD between urbanization and ecosystem services across scales in the YRB. U-A refers to urban agglomeration.

Spatially, the distribution of CCD at different scales significantly differs between the east and west, and north and south. Areas with severe county imbalance are concentrated in central Shanxi, Guanzhong Plain, Ningxia along the Yellow River, and the Lanzhou-Xining urban agglomeration, and generally spread in the upper and middle reaches of the Yellow River (Fig 4). Most counties in the central Shanxi Province are resource-based and have poor ecological environments. The Ningxia urban agglomeration along the Yellow River and Guanzhong Plain has a poor ecological environment. Due to its high ecosystem service index and low urbanization level, the Lanzhou Xining urban agglomeration suffers from a severe imbalance of the urbanization lag type. The mild disorder areas are mainly distributed at the edges of the severe disorder areas. They are concentrated in the Shandong Peninsula urban agglomeration, Central Plains urban agglomeration, and Huhhot-Baotou-Ordos-Yulin urban agglomerations. Moderately coordinated regions are distributed in the Shandong Peninsula, Central Plain, and Huhhot-Baotou-Ordos-Yulin urban agglomerations, with their number increasing in 2020. Notably, the Qinghai-Tibetan Autonomous Prefecture exhibits excellent coordination, showing a low level of synchronization and coordination due to low levels of urbanization and ecosystem services. The urbanization rates of Hang Jin Banner and Etoke Banner are 63.47% and 79.62%, respectively.

Next, the urbanization and ecosystem service levels of some counties and prefectures scattered in the coastal area of Shandong province are high, exhibiting high level and good coordination. Meanwhile, the areas with severe disorder at the prefecture level show the same spatial distribution characteristics as those at the county level. Moderately coordinated prefectures are concentrated in the Shandong Peninsula, Central Plain, and Huhhot-Baotou-Ordos-Yulin urban agglomerations. Seriously uncoordinated prefectures are concentrated in Ningxia along the Yellow River and Lanzhou Xining urban agglomeration, while moderately uncoordinated prefectures are scattered in the Guanzhong Plain urban agglomeration and concentrated in central Shanxi urban agglomeration. In 2019, the high-quality development plan for the YRB sought to rectify areas with low levels of coordination, paying more attention to ecological protection in the process of urbanization, and abandon polluting and consumptive industries to drive urban economic development. In 2020, the Shandong Peninsula and Central Plains urban agglomerations transformed from moderate to excellent coordination. These areas have always been in excellent coordination with solid stability. This shows that analyzing spatial changes from a macro perspective can better highlight the overall characteristics and changing trends.

The spatial distribution characteristics of the CCD at different scales differ. The CCD is more stable at the urban agglomeration scale than that at the prefecture and county scales, and the change is the smallest. However, the smaller the size, the stronger the regional heterogeneity. Notably, the urban agglomeration scale is in the stage of excellent coordination. Meanwhile, the prefecture and county scales exhibit moderate coordination or disorder. This shows that the overall coordination of urbanization and ecosystem services at the macro-scale does not prove that the internal prefecture–county system is also coordinated. Mesoscale prefectures and microscale counties show unique characteristics of urbanization development and ecosystem services. This may be because the macroscale perspective often ignores the existing defects in the urbanization and ecosystem service development process from the mesoscale or microscale perspective.

### 4.3 Spatial correlation characteristics of CCD between urbanization and ecosystem services in the YRB

Next, we measure the global spatial autocorrelation of the CCD between urbanization and ecosystem services from 2000 to 2020 and explore the spatiotemporal heterogeneity of the CCD. The spatial dependence of the CCD at all spatial scales in the YRB is significant, showing a positive spatial correlation. The Moran’s I values at multiple scales in 2020 are county (0.413) < prefecture (0.534) < urban agglomeration scales (0.670). The spatial aggregation of the CCD at all scales has weakened over time, indicating that the agglomeration effect of core cities has weakened.

**Fig 5:**
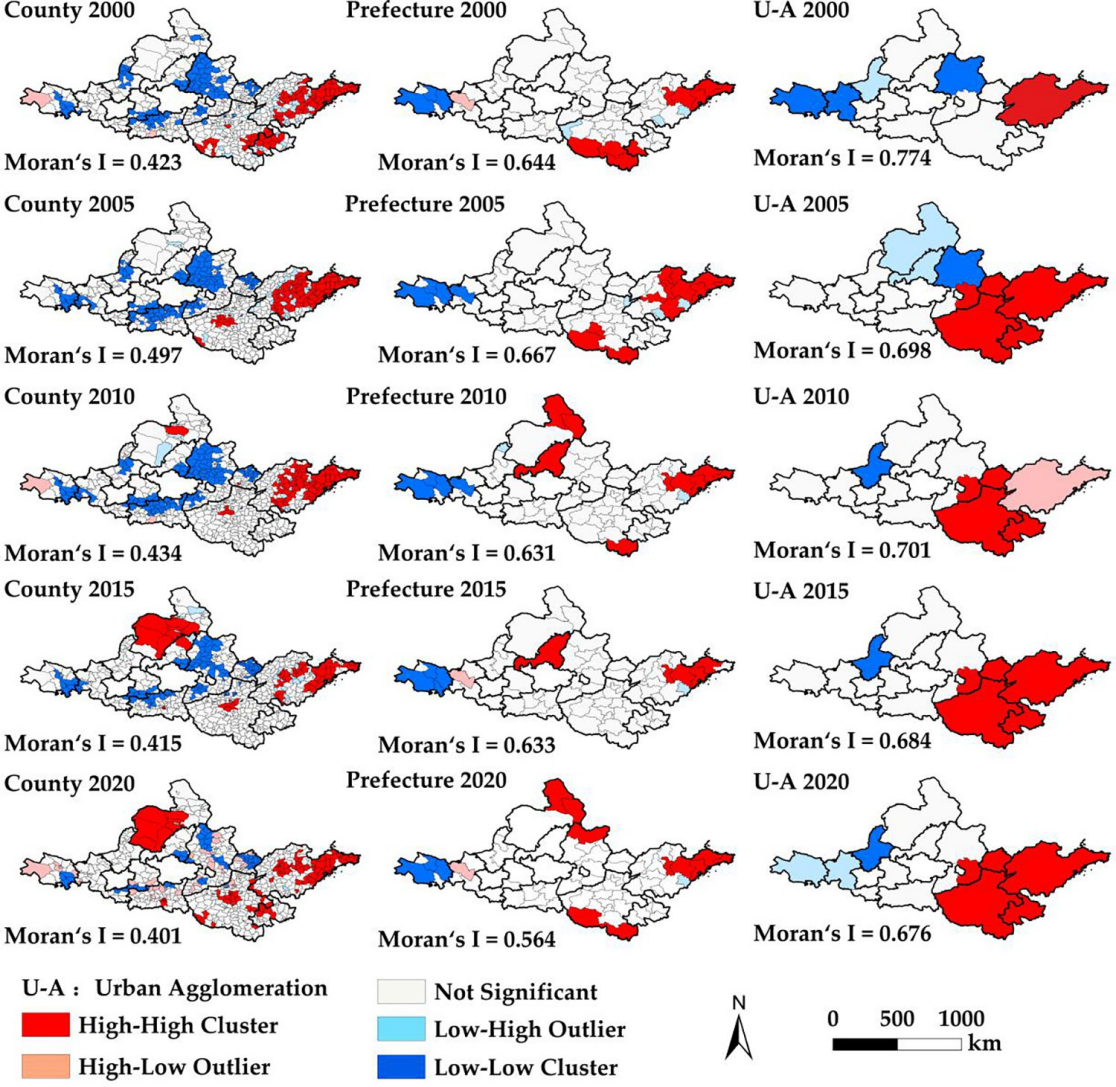
Distribution of spatial correlation types of CCD between urbanization and ecosystem services across scales in the YRB. U-A refers to urban agglomeration.

Next, we conduct a LISA agglomeration analysis, dividing spatial agglomeration categories into four regions: H-H, H-L, L-H, and L-L (Fig 5). From 2000 to 2020, all three scales show significant agglomeration changes, obvious polarization, and good spatial stability. The county-level H-H agglomeration has expanded from the central and eastern regions of the Shandong Peninsula to its whole prefectural group. In 2020, it spread to the urban agglomeration of the Shandong Peninsula, and the central and western regions of the Central Plain urban agglomeration. The H-H agglomeration-type area of Huhhot-Baotou-Ordos-Yulin urban agglomerations has expanded from Hangjin Banner to the county of Ordos. In 2015, Kundulun District and Tumed Right Banner of Baotou City were H-H agglomeration areas.

The L-L agglomeration type is dominant, accounting for 15.59–39.54% of the total number of county units, mainly distributed in the central Shanxi urban agglomeration, central and northern Ningxia urban agglomeration along the Yellow City agglomeration, Guanzhong Plain urban agglomeration, and the eastern counties of Lanzhou-Xining urban agglomeration. It is scattered in the central Shanxi urban agglomeration, northern edge of Guanzhong Plain urban agglomeration, and exhibits dot distribution in the middle of Lanzhou Xining urban agglomeration.

The H-L agglomeration areas are mainly distributed at the edge of the L-L agglomeration area, with slight spatial variation, with their numbers gradually increasing to 87 counties in 2020. From 2000 to 2015, L-L agglomeration prefectures were mainly distributed in areas with good development potential in the middle and lower reaches of the Yellow River. The high-quality coupling and coordination areas gradually became consistent with county and urban agglomeration scales. The spatial agglomeration area at the urban agglomeration scale shows little change. The H-H agglomeration area is mainly distributed in the Shandong Peninsula and Central Plains urban agglomerations. The L-L agglomeration area is distributed in central Shanxi and Ningxia along the Yellow River prefecture group, with little spatial change over time. The stability of spatial agglomeration is the strongest at multiple scales.

In summary, by 2020, H-H agglomeration areas at different scales are generally distributed in the Shandong Peninsula and Central Plains urban agglomeration, whereas L-L agglomeration areas are mainly distributed in central Shanxi, the Guanzhong Plain, and local areas of the Lanzhou Xining urban agglomeration. The larger the scale of the study area, the more macro-agglomerations are observed. The non-significant areas at the urban agglomeration scale show significant spatial agglomeration characteristics at the county and prefecture scales. This is an essential geographical basis for future research on targeted and personalized policy suggestions for urban development.

### 4.4 Driving mechanism of spatial pattern of CCD between urbanization and ecosystem services

#### 4.4.1 Selection of influencing factors and identification of leading factors

The explanatory variables for county, prefecture, and urban agglomeration scales are imported into the geographic detector model. The R program is used to select the optimal discrete classification method, and we obtain the influence q value of the explanatory variables at each scale on the CCD of the two systems in the YRB (**Table 5**).

**Table 5:** Results of geographic detector model on the influencing factors of CCD of the two systems at multiple scales in the YRB.

Driving factors	County *q* value						Prefecture *q* value						Urban agglomeration *q* value					
	2000	2005	2010	2015	2020	Order	2000	2005	2010	2015	2020	Order	2000	2005	2010	2015	2020	Order
Z_1_	0.15	0.25	0.26	0.38	0.24	1	0.02	0.36	0.51	0.39	0.52	6	0.10	0.28	0.39	0.26	0.52	10
Z_2_	0.14	0.14	0.21	0.22	0.17	5	—	0.30	0.37	0.47	0.62	4	0.95	0.93	0.96	0.93	0.89	3
Z_3_	0.16	0.16	0.17	0.21	0.17	6	—	0.33	0.44	0.51	0.54	3	0.95	0.96	0.96	0.92	0.93	2
Z_4_	0.10	0.31	0.13	0.25	0.12	4	—	0.37	0.29	0.25	0.21	8	0.48	0.53	0.59	0.70	0.62	8
Z_5_	0.18	0.23	0.19	0.19	0.23	3	—	0.38	0.20	0.45	0.26	7	0.97	0.96	0.99	0.92	0.93	1
Z_6_	0.08	0.15	0.12	0.18	0.16	8	—	0.19	0.21	0.20	0.25	9	0.76	0.80	0.83	0.94	0.85	6
Z_7_	0.11	0.14	0.12	0.19	0.23	7	—	0.55	0.47	0.54	0.74	1	0.72	0.95	0.88	0.94	0.88	5
Z_8_	0.08	0.08	0.10	0.16	0.20	9	—	0.48	0.43	0.38	0.56	5	0.76	0.80	0.83	0.82	0.88	7
Z_9_	0.15	0.19	0.23	0.28	0.22	2	—	0.55	0.43	0.41	0.71	2	0.76	0.95	0.98	0.92	0.85	4
Z_10_	0.07	0.10	0.09	0.08	0.18	10	—	0.17	0.1	0.1	0.37	10	0.53	0.09	0.18	0.62	0.04	11
Z_11_	—	—	—	—	0.11	11	0.02	0.19	0.11	0.1	0.14	11	0.51	0.31	0.33	0.41	0.32	9

Note: The Z_11_ index at the county scale only passes the test in 2020. The P value at the prefecture scale in 2000 is 0.421, which fails the test.

The mean explanatory power of the influencing factors across scales is as follows: county (0.11–0.26) < prefecture (0.13–0.55) < urban agglomeration scales (0.09–0.94). Economic development, social investment, and environmental protection factors play a leading role in the CCD of spatial patterns of multiscale urbanization and ecosystem services. Essential public services and government behavior play an important role, while natural factors have the smallest influence. The average slope of the western urban agglomeration in the YRB exceeds 10°, level of urbanization development is low, ecological damage is minor, and CCD is small. The eastern plain region has concentrated population, promising economic development, a good foundation for urban development and construction, high technology and environmental protection ability, and high coupling and coordination.

The dominant factors at each scale are different. Those at the county scale are GDP per capita, CO_2_ emissions, and total social fixed asset investment. The dominant factors at the municipal scale are CO_2_ emissions, road length, and the savings balances of urban and rural residents. The dominant factors of the urban agglomeration scale are the balance of urban and rural residents’ savings, investment in society’s fixed assets, and the proportion of the added value of secondary and tertiary industries in the GDP.

The factor detection results show that, as the spatial scale increases, the explanatory power of GDP and fiscal expenditure per capita gradually increases. In particular, the explanatory power of GDP per capita is ranked first at the county scale, sixth at the prefecture scale, and last at the urban agglomeration scale, indicating that the micro-influencing factors of per capita GDP have greater explanatory power at the micro-scale. The proportion of the added value of secondary and tertiary industries in the GDP, balance of savings of urban and rural residents, and investment in fixed assets of the whole society gradually rank at the top in terms of explanatory power as the scale increases. The industrial structure is an essential macro dimension for measuring economic development. Meanwhile, the balance of savings is an important indicator of the disposable income of residents and people’s living standards. Investment in the fixed assets of society further adjusts the economic structure and regional distribution of productive forces by establishing new sectors, enhancing economic strength, and creating material conditions for improving people’s material and cultural life. The three economic indicators and macro impact factors of social investment have excellent explanatory power at the urban agglomeration scale. The influencing factors with excellent explanatory power at the municipal scale include road length (national and provincial roads) and CO_2_ emissions. The spatial scale of the explanatory power of road length is as follows: prefecture (2^nd^) > urban agglomeration (4^th^) > county scales (7^th^). Dependence on transportation accessibility is significantly lower at the county scale than at the prefecture scale. As an essential factor for measuring environmental protection, CO_2_ emissions is a mesoscale factor and its explanatory power at the municipal scale is strong.

#### 4.4.2 Interactive detection results

**Fig 6:**
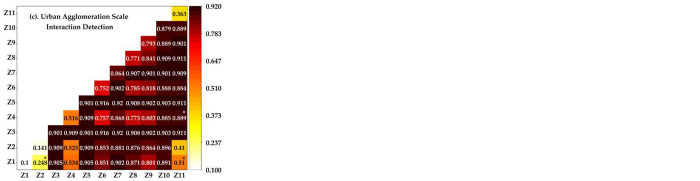
Interactive detection results of the driving mechanisms of the CCD between the multi-scale two systems in the YRB from 2000 to 2020. Note: The interaction in Fig 6(A) and 6(B) is nonlinear enhancement. In Fig 6(C), *denotes nonlinear enhancement and the rest is two-factor enhancement.

Each influencing factor has an interactive effect on the evolution process of the spatial pattern of the CCD between urbanization and ecosystem services at the county, prefecture, and urban agglomeration scales from 2000 to 2020. The explanatory power significantly increases after the pairwise interaction of different influencing factors. The multiscale interaction detection results show different degrees of two-factor or nonlinear enhancement without weakening or independent relationships (Fig 6).

The spatial pattern of the coupling relationship between multiscale urbanization and ecosystem services in the YRB is due to the interactions between economic development, government behavior, social investment, essential public services, environmental protection, and natural factors.

The interactions between CO_2_ emissions, GDP per capita, and other factors are strong at the county scale. The explanatory power is more than 44%, indicating that the development of the CCD spatial pattern of urbanization and ecosystem services in the YRB is mainly affected by economic development, environmental protection, and other factors. Next, the influence of the number of primary and secondary school students at the county scale is weak in single-factor detection. In contrast, interactive detection shows a nonlinear enhancement after interaction with other factors, especially when the q values of the interaction with GDP per capita and road length reach 0.849 and 0.682, respectively. Only the education level has a long and slow effect on the spatial pattern of CCD at the county scale. At the prefecture scale, the savings balance of urban and rural residents, road length, and CO_2_ emissions strongly interact with other factors, and the explanatory power q value exceeds 70%. The interactive detection of all factors at the prefecture scale shows nonlinear enhancement. The explanatory power of environmental protection, traffic accessibility, and government per capita fiscal expenditure is much higher than that of factor detection, peaking at 0.883. This shows that municipal government behavior, environmental factors, and fundamental public service policies significantly impact other factors, and their leading role is significant. The urban agglomeration scale’s q-value in the factor detection stage has the most substantial multiscale explanatory power, and interactive detection has the most interaction types enhanced by double factors. Among them, the proportion of per capita GDP and the added value of secondary and tertiary industries in the total added value, per capita GDP and average slope, and society’s fixed asset investment and average slope show nonlinear enhancement. The q values after the interaction are 0.248, 0.510, and 0.889, respectively. This shows that per-capita GDP is at the bottom of the scale factor detection of urban agglomeration, which needs to be fully reflected by the combined effects of macroeconomic influencing factors, social investment, and natural factors. Meanwhile, it reflects that adjusting the social investment level has a certain effect. The transformation of economic structure plays a significant role in improving people’s living standards. Economic and ecological sustainable development policies, such as strengthening environmental protection and optimizing the economic structure. This is conducive for improving the CCD between urbanization and ecosystem services in the YRB to excellent coordination.

#### 4.4.3 Formation mechanism of the coupling coordination relationship between urbanization and ecosystem services

**Fig 7:**
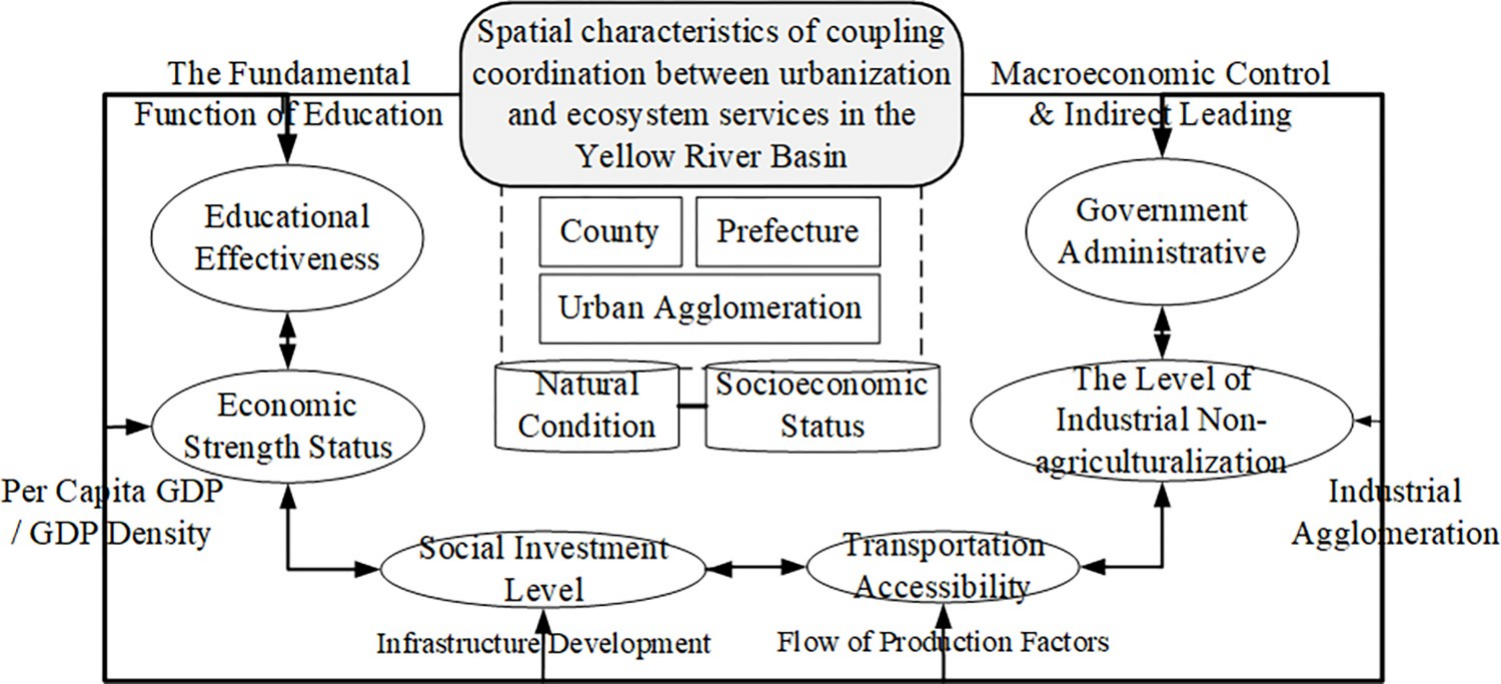
Formation mechanism of the spatial pattern of CCD between multi-scale urbanization and ecosystem services in the YRB.

Based on the results of dominant factor identification and interaction detection, we construct the formation mechanism of the spatiotemporal evolution of the CCD between multiscale urbanization and ecosystem services in the YRB (Fig 7).

The explanatory power of GDP per capita and government fiscal expenditure per capita at the county scale is substantial, reaching 0.43, and interaction detection with other factors shows nonlinear enhancement. The CCD between urbanization and ecosystem services at the county scale is directly driven by GDP per capita and government behavior. The explanatory power of road length and CO_2_ emissions at the municipal scale is strong, reaching the highest value of 0.74. Traffic accessibility and environmental protection are necessary to promote the flow of production factors and enhance the sustainable development of the economy, society, and ecology, directly affecting the CCD of urbanization and ecosystem services at the municipal level. At the urban agglomeration scale, society’s fixed asset investments, and the savings balance of urban and rural residents have strong explanatory power.

In conclusion, the dominant factors influencing the spatial pattern of the CCD between multiscale urbanization and ecosystem services in the YRB are different. The dominant influencing factors at the county scale are micro-influencing factors directly related to people’s living standards and quality. At the prefectural scale, the meso-level enhances the livability characteristics of the prefecture, which can ensure the overall urban and rural living standards and quality of influencing factors. Thus, considering the economic and industrial structures, and social asset investment, the CCD between urbanization and ecosystem services in the YRB is comprehensively affected by various factors, such as the economy, government policy, and environmental protection, at the micro, meso, and macro levels.

## 5 Discussion

Significant spatial and temporal differences exists in the level of multiscale urbanization and ecosystem service capacity in the YRB from 2000 to 2020. This study discusses the spatial distribution characteristics of urbanization and ecosystem services in the YRB at different spatial scales. The city’s mesoscale and county’s microscale show different system-level distributions from the macroscale of the urban agglomeration. This indicates that the larger the scale, the more macro the research perspective, and the results show strong integrity characteristics. Among the characteristics of the system’s overall high/low level, there are heterogeneous characteristics within the prefecture and county. By comparing the development levels of urbanization and ecosystem services from different spatial perspectives, we make reasonable suggestions to compensate for the unbalanced development of county urbanization and protect the ecological environment [41].

Analyzing the spatiotemporal characteristics at different scales, the subsystem development level of the two systems is closely related to the CCD level of the whole system. The larger the scale, the higher the degree of CCD. We find an upward trend in the coupling coordination at various scales. However, the overall level is not high, and mainly manifests as a transition from moderate imbalance to moderate coordination. The intra-regional differences across scales are ordered as follows: urban agglomeration < prefecture < county. This is consistent with prior research [37]. We highlight the stages and heterogeneity of the coupling coordination of the two systems in the multi-scale coupling and coordination development of urban agglomerations, prefectures, and counties. Further, we provide theoretical explanations for the severe polarization phenomenon and intensification of regional imbalance. We propose a geographical basis for future research and development of government policies, and fundamental changes in the coupling relationship between urbanization and ecosystem services.

Based on multiscale CCD driving mechanism analysis, Wang and Xu [39] mainly referred to the principle and prospect of geographical detectors. Importantly, we find that the larger the scale, the greater the explanatory power of the influencing factors. However, differences in spatial scale lead to different dominant influencing factors. Per capita economic and financial indicators have a more significant impact on county-scale CCD, while environmental protection and transportation factors have a more significant impact on prefecture-scale coupling and coordination [42]. Economic structure, social investment, and other factors have a more significant impact on the urban agglomeration scale. Different economic indicators also have different explanatory power at different spatial scales. This differs from the research results driven by a single spatial scale economic factor [43, 44]. Further, we find that regions with high economic development levels, reasonable industrial structures, developed infrastructure, and better environmental governance have more resource allocation advantages and promote system coupling and coordination development, consistent with Li and Zhang [16].

This study has some limitations. First, the statistical caliber of the relevant data in the different regions was somewhat consistent. Second, we have only conducted our analysis using an existing index system, which may lead to errors in the conclusions. Third, we have considered each unit of the administrative region within the county, prefecture, and urban agglomeration scales as an independent research object while exploring the level of CCD. Future works can integrate multi-source remote sensing data to enrich the rationality and reliability of the demonstration data. Further, owing to data availability concerns, there is no long time series for continuous years of research; thus, researchers should confuct precise and in-depth research.

At the urban agglomeration scale, national macro policy, fiscal revenue, and expenditure policy, such as gross national product, industrial added value, and other macro indicators, can be used to guide the overall direction of high-quality urban development and ecological protection in the YRB.

At the county level, stakeholders and policymakers can focus on specific micro dimensions, such as per capita GDP, and per capita fiscal revenue and expenditure. For good urban agglomeration development, local underdeveloped counties should undertake targeted development efforts focused on the key influencing factors at the county scale. Further, specific methods of county urbanization and ecological protection can be formulated according to the county’s regional characteristics.

At the prefecture scale, spatial policy planning should be conducted from the meso-dimensions of the degree of urban greening, urban construction, urban environmental protection, and overall urban and rural development to create a good link and bridge between the macro perspective of urban agglomerations and the micro perspective of counties. Efforts can improve urban priorities, and promote the high-quality development of urbanization and ecological protection of urban agglomerations in flow regions.

## 6 Conclusion

Promoting the coupled and coordinated development of urbanization and ecosystem services is essential for sustainable development. Considering the multi-spatial scales in the YRB, this study uses spatial autocorrelation analysis to explore the spatial evolution characteristics of the coupled and coordinated development of multiscale urbanization and ecosystem services in the YRB from 2000 to 2020. We use geographic detectors to identify the main influencing factors and their interactions. Our findings are outlined below.

First, the spatiotemporal differences in the CCD between urbanization and ecosystem services at different scales are significant and show an upward trend. The larger the scale, the greater the CCD. The overall distribution characteristics of the CCD at the county, prefecture, and urban agglomeration scales are consistent, showing that the east is high and the west is low. However, the county and prefecture regions show noticeable regional particularities: the area with high coupling of the entire urban agglomeration, and the local area with low CCD of the inner prefecture and county of the urban agglomeration.

Second, we find a significant positive spatial correlation between the CCD at each spatial scale. The polarization characteristics of the CCD at different scales are significant with good spatial stability. The larger the scale, the stronger the agglomeration effect. Over time, the spatial agglomeration of the CCD gradually weakens and the agglomeration effect of core cities also weakens. The local characteristics of county-scale agglomerations are opposite to those of prefectures and urban agglomerations. The smaller the scale, the more specific the particularities.

Finally, the influencing factors differ across scales. The larger the scale, the stronger the explanatory power of the influencing factors. The interactive probing results show varying degrees of two-factor or nonlinear enhancement. Natural and socioeconomic factors drive the spatial pattern of the CCD; the natural conditions have gradually weakened and socioeconomic conditions have gradually strengthened.

## Supporting information

S1 Data (ZIP)

S1 File (RAR)

## References

[1] Liu Z, He C, Zhou Y, et al. How much of the world’s land has been urbanized, really? A hierarchical framework for avoiding confusion[J]. Landscape Ecology, 2014, 29(5).

[2] Dou Y, Zhen L, Yu X, et al. Assessing the influences of ecological restoration on perceptions of cultural ecosystem services by residents of agricultural landscapes of western China[J]. Science of the Total Environment, 2019, 646. 30059928 10.1016/j.scitotenv.2018.07.205

[3] Christopher D B, Femke R, Giovanni B, et al. Future urban land expansion and implications for global croplands.[J]. Proceedings of the National Academy of Sciences of the United States of America, 2017, 114(34). 10.1073/pnas.1606036114 PMC5576776 28028219

[4] Carlson N T, Arthur T S. The impact of land use—land cover changes due to urbanization on surface microclimate and hydrology: a satellite perspective[J]. Global and Planetary Change, 2000, 25(1).

[5] Chi C, Taejin P, Xuhui W, et al. China and India lead in greening of the world through land-use management.[J]. Nature sustainability, 2019, 2(2). 10.1038/s41893-019-0220-7 PMC6376198 30778399

[6] Zhongwu Z, Jinyuan Z, Liping L, et al. Spatial–Temporal Heterogeneity of Urbanization and Ecosystem Services in the Yellow River Basin[J]. Sustainability, 2023, 15(4).

[7] Tianqi R, Pengyan Z, Guanghui L, et al. Spatial correlation evolution and prediction scenario of land use carbon emissions in the Yellow River Basin[J]. Ecological Indicators, 2023, 154.

[8] Tianyu Z, Haimeng L, Peng G, et al. Conflict or Coordination? measuring the relationships between urbanization and vegetation cover in China[J]. Ecological Indicators, 2023, 147.

[9] Zhuonan H, Yingbiao C, Zihao Z, et al. Spatiotemporal coupling analysis between human footprint and ecosystem service value in the highly urbanized Pearl River Delta urban Agglomeration, China[J]. Ecological Indicators, 2023, 148.

[10] Yin K, Xu Y, Li X, et al. Sectoral relationship analysis on China’s marine-land economy based on a novel grey periodic relational model[J]. Journal of Cleaner Production, 2018, 197.

[11] Jing J, Sisi Z, Hailong L, et al. A design-support framework to assess urban green spaces for human wellbeing[J]. Sustainable Cities and Society, 2023, 98.

[12] Chuanglin F, Haimeng L, Shaojian W. The coupling curve between urbanization and the eco-environment: China’s urban agglomeration as a case study[J]. Ecological Indicators, 2021, 130.

[13] Liu H, Fang C, Fang K. Coupled Human and Natural Cube: A novel framework for analyzing the multiple interactions between humans and nature[J]. Journal of Geographical Sciences, 2020, 30(4).

[14] Wei L, Jinyan Z, Fen Z, et al. Spatio-temporal variations of ecosystem services and their drivers in the Pearl River Delta, China[J]. Journal of Cleaner Production, 2022, 337.

[15] Jianxin Xiong, Xinbin Wang, Di Zhao, Yayuan Zhao. Spatiotemporal pattern and driving forces of ecological carrying capacity during urbanization process in the Dongting Lake area, China[J]. Ecological Indicators, 2022, 144.

[16] Chengyu Li, Shiqiang Zhang, 2020. Chinese provincial water-energy-food coupling coordination degree and influencing factors research. China Population, Resources and Environment.30(1): 120–128.

[17] Qi Zhang et al. [Analysis on the coordinated development of ecology-economy-society in coal resource cities: A case study of Huainan, China].[J]. The journal of applied ecology, 2019, 30(12): 4313–4322. 10.13287/j.1001-9332.201912.012 31840478

[18] Shiwang Yu, Qi Zhang, Hao Jian Li Ma Wenting, Yao Sun, Xuechao Wang, et al. Development of an extended STIRPAT model to assess the driving factors of household carbon dioxide emissions in China[J]. Journal of Environmental Management, 2023, 325(PA). 10.1016/j.jenvman.2022.116502 36274310

[19] Xiangxi Kong, Wenjie Li, Jiao Jiang, Zhixu Dong, Zhaozhi Wang. Dynamic characteristics of a simply supported elastic beam with three induction motors[J]. Journal of Sound and Vibration, 2022, 520.

[20] Can Yang, Tianxing Wei, Yiran Li. Simulation and Spatio-Temporal Variation Characteristics of LULC in the Context of Urbanization Construction and Ecological Restoration in the Yellow River Basin[J]. Sustainability, 2022, 14(2).

[21] Wenjie Wu, Huijie Li, Hao Feng, Bingcheng Si, Guangjie Chen, Tingfang Meng, et al. Precipitation dominates the transpiration of both the economic forest (Malus pumila) and ecological forest (Robinia pseudoacacia) on the Loess Plateau after about 15 years of water depletion in deep soil[J]. Agricultural and Forest Meteorology, 2020(prepublish).

[22] Jinyu Hu, Jiaxin Zhang, Yunqin Li. Exploring the spatial and temporal driving mechanisms of landscape patterns on habitat quality in a city undergoing rapid urbanization based on GTWR and MGWR: The case of Nanjing, China[J]. Ecological Indicators, 2022, 143.

[23] Yuhang G, Shiyuan F, Haitang C, et al. Identifying the spatio-temporal pattern of drought characteristics and its constraint factors in the Yellow River Basin[J]. Ecological Indicators, 2023, 154.

[24] Peng W, Mingxiang X. Dynamics and interactions of water-related ecosystem services in the Yellow River Basin, China[J]. Journal of Geographical Sciences, 2023, 33(8).

[25] Shuai C, Xiuying W, Shunbo Y. National water-saving city and its impact on agricultural total factor productivity: A case study of nine provinces along the Yellow River, China[J]. Journal of Cleaner Production, 2023, 417.

[26] Menghao Y, Xiaodong G, M H K S, et al. Spatiotemporal exploration of ecosystem service, urbanization, and their interactive coercing relationship in the Yellow River Basin over the past 40 years.[J]. The Science of the total environment, 2022, 858(Pt 1). 10.1016/j.scitotenv.2022.159757 36349629

[27] Wei L, Jinyan Z, Fen Z, et al. Exploring the coupling relationship between urbanization and energy eco-efficiency: A case study of 281 prefecture-level cities in China[J]. Sustainable Cities and Society, 2021, 64.

[28] Jin Hui Zhang Xiu Fen Shi. Application of Variation Coefficient Method and TOPSIS Model on Urban Environmental Quality Assessment[J]. Key Engineering Materials, 2010, 930(439–440).

[29] Yangcheng Hu, Yi Liu, Zhongyue Yan. Research Regarding the Coupling and Coordination Relationship between New Urbanization and Ecosystem Services in Nanchang[J]. Sustainability, 2022, 14(22).

[30] Ardavan Zarandian, Fatemeh Mohammadyari, Mirsanjari Mir Mehrdad Visockiene Jurate Suziedelyte. Scenario modeling to predict changes in land use/cover using Land Change Modeler and InVEST model: a case study of Karaj Metropolis, Iran[J]. Environmental Monitoring and Assessment, 2023, 195(2). 10.1007/s10661-022-10740-2 36607450

[31] Rustam M, Nenad M, Bassem J, et al. How to Use K-means for Big Data Clustering?[J]. Pattern Recognition, 2023, 137.

[32] Li Zhao, Mengwei Su, Xueyan Wang. Spatial–Temporal Evolution and Prediction of Habitat Quality in Beijing–Tianjin–Hebei Region Based on Land Use Change[J]. Land, 2023, 12(3).

[33] Donghui Shi, Qiusheng Wu, Yu Li. Multidimensional assessment of soil conservation ecosystem services and multiscale analysis of influencing mechanisms[J]. Journal of Cleaner Production, 2022, 381(P1).

[34] Steiner Jean L., Devlin Daniel L., Technology, and Management Options for Water Conservation in the Ogallala Aquifer in Kansas, USA[J]. Water, 2021, 13(23).

[35] Yixuan Yang, Shiqin Zhang, Fan Xia, Yixuan Yang, Dehuan Li, Wei Sun, et al. A comprehensive perspective for exploring the trade-offs and synergies between carbon sequestration and grain supply in China based on the production possibility frontier[J]. Journal of Cleaner Production, 2022, 354.

[36] Ecology Research—Wetland Ecology; Study Findings from J.C. Merriman et al Provide New Insights into Wetland Ecology (Rapid ecosystem service assessment of the impact of Koshi Tappu Wildlife Reserve on wetland benefits to local communities)[J]. Ecology, Environment & Conservation, 2018.

[37] Dengyu Yin, Haochen Yu, Yanqi Lu, Jian Zhang, Gensheng Li, Xiaoshun Li. A Comprehensive Evaluation Framework of Water-Energy-Food System Coupling Coordination in the Yellow River Basin, China[J]. Chinese Geographical Science, 2023, 33(2). 10.1007/s11769-023-1344-2 PMC10033299 36974306

[38] DeWitt T. J., Fuentes J. I., Ioerger T. R., Bishop M. P. Rectifying I: three point and continuous fit of the spatial autocorrelation metric, Moran’s I, to ideal form[J]. Landscape Ecology, 2021(prepublish).

[39] Jinfeng Wang, Chengdong Xu. Geographic detectors: principles and prospects [J]. Journal of Geography, 2017, 72 (01): 116–134.

[40] Peng H, RuTian B, LiShuai X, et al. [Using geographical detection to analyze responses of vegetation growth to climate change in the Loess Pla-teau, China].[J]. Ying yong sheng tai xue bao = The journal of applied ecology, 2022, 33(2). 35229519 10.13287/j.1001-9332.202202.012

[41] Zheneng H, Jianjiao G, Jiaxi L, et al. Valuing the coordinated development of urbanization and ecosystem service value in border counties[J]. Journal of Cleaner Production, 2023, 415.

[42] Haitao Ma. Urbanization under globalization: How does the Belt and Road Initiative affect urbanization levels in participating countries[J]. Journal of Geographical Sciences, 2022, 32(11).

[43] Jiatian Zhang. Characteristics of coordination changes and spatial coupling relationship between urbanization and ecosystem services[J]. Acta Ecologica Sinica, 2020, 40(10).

[44] Guo X, Fang C, Mu X, et al. Coupling and coordination analysis of urbanization and ecosystem service value in Beijing-Tianjin-Hebei urban agglomeration[J]. Ecological Indicators, 2022, 137.

